# A Treatise on Diseases of the Eye

**Published:** 1910-12

**Authors:** 


**A Treatise on Diseases of the Eye.** By **John E. Weeks,** M.D., Professor of Ophthalmology in the University and Bellevue Hospital Medical College, New York. Octavo, pp. 944, with 528 illustrations and 25 full-page plates. Lea & Febiger, Publishers, Philadelphia and New York. 1910. (Cloth, $6.00, net.)

The reception accorded this new book has been most flattering. It would be difficult for the author to write an uninstructive book, and impossible for him to write an uninteresting one. Here we have one of the most complete one-volume textbooks before the profession. Not only is there a full description of the anatomy of the eye, and the principles of optics, but the clinical aspect is also discussed in a clear and comprehensive manner. Due weight has been given to the recent developments in ophthalmology, including the application of modern methods of treatment. As little theory as possible has been given and only when known facts are not sufficient to explain conditions described. The chapters on refraction and special remedies alone are worth the price of the book. J. A. R.

